# Conditional cash incentives, community health workers, and continuum of maternal and child healthcare: evidence from India

**DOI:** 10.1093/heapol/czag019

**Published:** 2026-03-03

**Authors:** Nisha Mishra, Sukumar Vellakkal

**Affiliations:** Department of Economic Sciences, Indian Institute of Technology Kanpur, Kalyanpur, Kanpur District, Uttar Pradesh 202816, India; Department of Economic Sciences, Indian Institute of Technology Kanpur, Kalyanpur, Kanpur District, Uttar Pradesh 202816, India

**Keywords:** conditional cash transfer, community health workers, maternal and child healthcare, continuum of care, India, H51, H53, I15, I18

## Abstract

Continuum of care in maternal and child health (MCH) services is a key strategy for improving MCH outcomes. This study examines the effect of conditional cash incentives and community health worker support on the uptake of the continuum of MCH care, defined as the sequential utilization of antenatal, skilled delivery, and postnatal services. Using nationally representative cross-sectional datasets and a difference-in-difference framework, we find that both interventions significantly improved the continuum of MCH care. The intent-to-treat estimates showed a 5-percentage-point increase in the proportion of women completing the full continuum of care. Heterogeneity analysis revealed more substantial effects among educated women, those in urban areas, and those in higher wealth quintiles. Insights from qualitative interviews with mothers and community health workers suggested that awareness of antenatal care and institutional delivery increased; however, postnatal care was typically sought only in response to complications, and the uptake of all recommended MCH services as a full continuum was often hindered by intersecting demand- and supply-side barriers. Notably, participants emphasized that sustained community health worker engagement had a more significant impact on ensuring care continuity than cash incentives alone. These findings highlight the need for policy strategies that enhance community health worker-led support mechanisms, combined with financial incentives, to promote the comprehensive and sustained use of maternal health services among disadvantaged population groups.

Key messages

- Improving maternal and child health outcomes requires policies that treat the continuum of care as an integrated pathway across pregnancy, childbirth, and the postnatal period.
- Programs that pair demand-side financial incentives with sustained community health worker engagement demonstrate the value of integrated demand- and supply-side strategies to strengthen continuity of care.
- Persistent socioeconomic and geographic disparities in uptake of continuum-of-care services underscore the need for equity-oriented program design and targeted community outreach.
- Relationship-based engagement by community health workers, complemented by financial incentives, is critical to sustaining continuity of care.
- Long-standing national programs must continue addressing enduring socioeconomic and cultural barriers through strengthened community outreach and service readiness.

## Introduction

Despite efforts to reduce global maternal mortality, the rate remains high, with 223 maternal deaths per 100 000 live births in 2020, far from the SDG target of 70 by 2030. Maternal and child health (MCH) outcomes are poor in low- and middle-income countries (LMICs), with India accounting for 8.3% of global maternal deaths (WHO 2023). While India's under-five mortality rate has decreased from 127 per 1000 live births in 1990 to 31 in 2021, further reductions are needed to meet the SDG target of 25 by 2030 (WHO 2023). Although improvements in maternal and child mortality have been slow and uneven (Sen et al. 2020), a substantial share of maternal morbidity and mortality is preventable with improved access to MCH services, such as antenatal care (ANC), institutional delivery, and postnatal care (Bhutta et al. 2014, Okeke and Abubakar 2020). A continuum of care (CoC) in MCH links services across the life course—from the mother’s adolescence, pregnancy, childbirth, and postnatal period to the child’s early childhood—so that women and children receive timely, sequential contacts with health providers at each stage and are less likely to drop out between antenatal, delivery, postnatal, and early childhood services, thereby improving maternal and child health outcomes by reducing preventable morbidity and mortality (WHO 2005).

This paper evaluates the impact of conditional cash incentives and community health worker (CHW) support on achieving the CoC in MCH services among disadvantaged populations in India. Previous studies suggest that supply- and demand-side interventions, such as cash incentives and CHW support, can enhance CoC uptake (Batura et al. 2019, Ochieng et al. 2019). In India, the Janani Suraksha Yojana (JSY) program, which provides conditional cash incentives along with support from CHWs, called Accredited Social Health Activists (ASHAs), has contributed to increased institutional deliveries and improved maternal and child mortality outcomes (Lim et al. 2010, Carvalho et al. 2014, Joshi and Sivaram 2014, Powell-Jackson et al. 2015). Lim et al. (2010) found that participation in the JSY was associated with significant improvements in maternal and neonatal health outcomes, including increases of 11 and 49 percentage points in the utilization of antenatal care (ANC) and institutional deliveries, respectively, and reductions of 4.1 perinatal and 2.4 neonatal deaths per 1000 live births. These findings were later corroborated by Carvalho et al. (2014), who demonstrated that the results remained robust across alternative model specifications. In contrast, Powell-Jackson et al. (2015) reported a 7.5 percentage point increase in facility births following the introduction of JSY. However, they found no strong evidence of improvements in ANC uptake or reductions in neonatal mortality. Using an intent-to-treat approach, Joshi and Sivaram (2014) provided causal evidence that institutional deliveries increased by 3 percentage points. However, the gains were primarily concentrated among women from socially disadvantaged households. A recent study found that combining conditional cash incentives with ASHA support improved short-term birth outcomes, reporting a 5.1 percentage-point increase in institutional deliveries (Baulia 2023). However, there is limited research on access to the continuum of MCH services in India, with only a few studies addressing this gap (McDougal et al. 2017). Most existing research focuses on individual characteristics, such as women's education, wealth, and autonomy, as factors affecting CoC uptake (Shibanuma et al. 2018; Kothavale and Meher 2021).

Situated within the growing literature on conditional incentives and the role of CHWs in improving access to MCH services and outcomes in LMICs, particularly in India, this study offers three specific contributions that extend existing evidence. First, while previous research has primarily focused on individual outcomes such as institutional delivery, ANC, and PNC (Lim et al. 2010, Gopalan and Varatharajan 2012, Carvalho et al. 2014, Joshi and Sivaram 2014, Powell-Jackson et al. 2015, Rahman and Pallikadavath 2018, Debnath 2021), this study examines the program’s impact across the full continuum of maternal and child healthcare, encompassing four key services: ANC, institutional delivery, PNC, and child immunization. Second, in contrast to studies based on early-phase data from 2006–07, when ASHA coverage was effectively nonexistent and program implementation was still nascent despite its formal launch in 2005 (Das et al. 2011, Aizawa 2022), our analysis draws on data from the program’s mature phase (post-2012), when ASHAs had become an integral part of service delivery. Third, while most existing studies estimate average treatment effects on the treated (ATT), we estimate intent-to-treat (ITT) effects to capture the program impact across the broader target population, including eligible women who may not have fully utilized JSY benefits. This approach is particularly relevant given that, even after the targeted complete implementation phase in 2012, fewer than half of the eligible beneficiaries accessed the JSY cash transfer and ASHA support (Singh and Vellakkal 2023).

Using a difference-in-differences framework, we exploit variation across treatment and control groups to estimate the ITT effect of cash incentives and ASHAs on the CoC. The treatment and control groups have been defined based on the program's eligibility criteria. Under JSY eligibility rules, the treatment group comprises all pregnant women residing in low-performing states^1^ (LPS), as well as pregnant women from households below the poverty line (BPL) or designated backward caste households in high-performing states (HPS). The control group comprises ineligible pregnant women in HPS. We complement our quantitative analysis with qualitative interviews with mothers and community health workers to gain a deeper understanding of the factors hindering CoC access.

We organize the remainder of the paper as follows: Background section provides the program background, Mechanism section outlines the mechanism, Methods section describes the data and empirical methods, Results section presents the results, Discussion section provides discussion, and Conclusion section concludes.

## Background

### Janani Suraksha Yojana (JSY)

JSY, launched in April 2005 under the National Health Mission (NHM), is an ongoing national maternal health program aimed at reducing maternal and neonatal mortality by promoting institutional delivery among poor and disadvantaged women. The objective of JSY is to increase the uptake of the CoC in MCH services among disadvantaged pregnant women, thereby improving maternal and child health outcomes. The program combines two key components: a demand-side intervention offering conditional cash incentives for childbirth in healthcare facilities, and a supply-side intervention providing support from ASHAs. While JSY primarily targets increasing institutional delivery, it also encourages the uptake of antenatal services (e.g. Tetanus injections and iron-folic acid supplements) and at least one postnatal visit by incentivizing ASHAs (MoHFW 2006MoHFW accessed 20th April 2025).

ASHAs, village-based CHWs, play a critical role in connecting women to healthcare services. They are incentivized to register eligible women, provide them with pregnancy counseling, and ensure their attendance at health centers for delivery and PNC. ASHAs accompany beneficiaries to health centers, schedule postpartum appointments, and arrange for newborn immunizations. Their incentives depend on ensuring adequate ANC, skilled birth attendance, and PNC (Joshi and Sivaram 2014, Rahman and Pallikadavath 2018).

The program categorizes states into low-performing states (LPS) and high-performing states (HPS) based on their institutional birth rates (MoHFW 2006MoHFW accessed 20th April 2025). Eligibility and cash incentives differ across these categories and between rural and urban areas. In LPS, all pregnant women are eligible for cash incentives. In contrast, in HPS, eligibility is limited to women aged 19 or older from households below the poverty line (BPL) or from scheduled caste/scheduled tribe categories, with a cap of two live births per mother. Cash incentives for mothers vary; in LPS, rural mothers receive ₹1400 (approximately US$17), and urban mothers receive ₹1000 (approximately US$12); in HPS, rural mothers receive ₹700, and urban mothers receive ₹600. ASHAs receive ₹600 in rural areas and ₹400 in urban areas per childbirth, disbursed in two installments: after delivery and after the postnatal visits, and upon the child's immunization with Bacillus Calmette-Guérin (BCG).

## Mechanism

**Figure 1:**
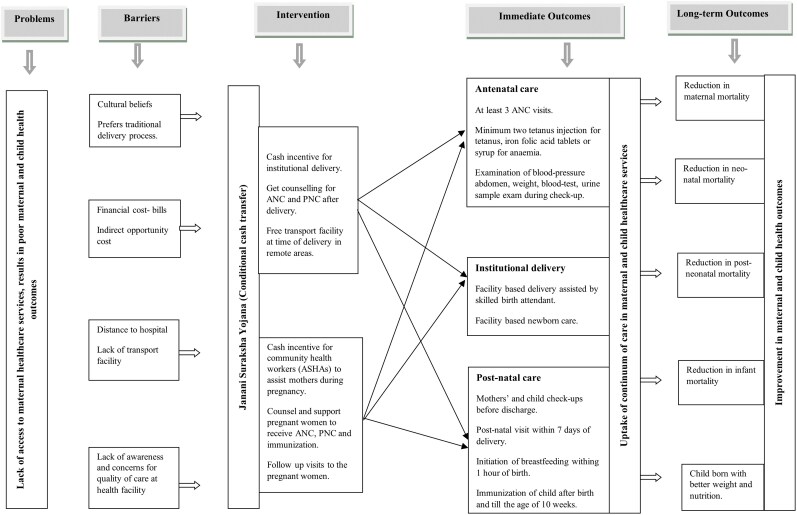
Causal pathways of JSY on the uptake of the continuum of maternal and child health care.

We outline the causal pathways linking cash transfers and community health workers to CoC in MCH services, as well as their impact on maternal and child health outcomes (Fig. 1).

### Phase 1: Rationale for conditional cash transfer intervention

Most maternal and child deaths are often due to limited access to healthcare, with barriers such as sociocultural factors, financial constraints, and lack of knowledge on hygiene and safety (Koblinsky et al. 2016, Vellakkal et al. 2017). Financial barriers include hospital charges and the associated opportunity costs. These costs affect optimal health demand, as individuals must trade off their time and resources for health (Grossman 2017). Rural women, especially those with limited education, often prefer home deliveries due to cultural beliefs and limited access to transportation to hospitals (Mistry et al. 2009, Zaveri et al. 2023). To address these demand- and supply-side barriers, JSY adopted a dual strategy. On the demand side, pregnant women received cash incentives conditional on their use of ANC services and on delivery assisted by a skilled birth attendant. On the supply side, village-based CHWs, known as ASHAs, counseled and assisted pregnant women during pregnancy.

### Phase 2: CoC uptake and maternal and child health outcomes

Conditional cash transfers have been increasingly used to encourage healthy behavior change. According to the incentive theory in behavioral economics, human behavior is driven by incentives or rewards, as individuals maximize their economic returns by timing their receipt (O’Donoghue and Rabin 1999, Katare 2021). The cash incentive provides immediate rewards for healthy behavior by compensating for financial and opportunity costs (Giles et al. 2014, Ochieng et al. 2019). We hypothesize that cash incentives and ASHA support increase hospital visits for ANC, childbirth, and PNC services. Cash incentives encourage them to take up services, and ASHA serves as a bridge between mothers and health facility stakeholders through awareness-raising and support services. The counseling sessions by ASHA to expectant mothers on the need for safety and hygiene during delivery help in shaping women`s perceptions regarding institutional delivery care and bring behavioral change. Mothers are more likely to reach institutional delivery care facilities on time when ASHAs help arrange transportation facilities and accompany them during delivery. Periodic home visits by ASHAs can ensure that pregnant women attend at least 3 ANC checkups, receive tetanus injections and iron–folic acid (IFA) tablets, and undergo PNC checkups after delivery. This leads to improved CoC retention and, over time, better health outcomes, including reduced maternal and child mortality and improved birth weight (Singh and Vellakkal 2021, Usman et al. 2021).

## Materials and methods

### Secondary data for quantitative analysis

#### Data and variable descriptions

We utilize repeated, individual-level cross-sectional data from the National Family Health Survey (NFHS). Specifically, we use the NFHS-2 (1998–99) as the preprogram period and NFHS-4 (2015–16) as the postprogram launch period. The NFHS is a nationally representative survey that provides detailed information on fertility, mortality, reproductive and child health, family welfare, and nutrition, along with household, demographic, and socioeconomic characteristics, including caste, religion, wealth, and poverty status. These two waves are suitable for this study, as NFHS-2 predates the program's launch by several years, while NFHS-4 was conducted nearly a decade after its implementation. NFHS-2 interviewed 90 303 ever-married women aged 15–49 years across 90 486 households, while NFHS-4 surveyed 699 686 women in the same age group across 601 509 households throughout India.

Since NFHS-2 reports mother-and-child-level data only for children under three, to ensure comparability, we restricted the sample to women’s most recent births within the past three years in both survey rounds. We identified women eligible for JSY based on their state of residence, caste, age at birth, and poverty status. Although JSY was implemented nationally, to ensure a clean comparison group not exposed to concurrent state-level maternity benefit schemes, we excluded four states—namely, Madhya Pradesh, Maharashtra, Odisha, and Tamil Nadu—that operated such programs during the same period.^2^ We also excluded women with more than seven children to minimize the influence of outliers, and removed respondents from six union territories that were not surveyed in NFHS-2. The final analytical sample comprised 126 455 ever-married women, including 21 603 from NFHS-2 and 104 852 from NFHS-4. Although the analytical sample is more heavily drawn from NFHS-4, reflecting its larger overall survey size, the NFHS-2 sample remains statistically adequate for the analysis. Because the outcome variables relate to access to MCH services—specifically the CoC—measured among women of reproductive age, the minimum required sample for robust estimation is relatively modest. To confirm adequacy, we estimated the minimum required sample size for each outcome variable and conducted a *post hoc* power analysis using Stata. The results indicated that the minimum required sample size for NFHS-2 ranged between 1000 and 1500 across different outcome variables, while the achieved power values were close to 1.0, confirming sufficient statistical precision.

**Figure 2:**
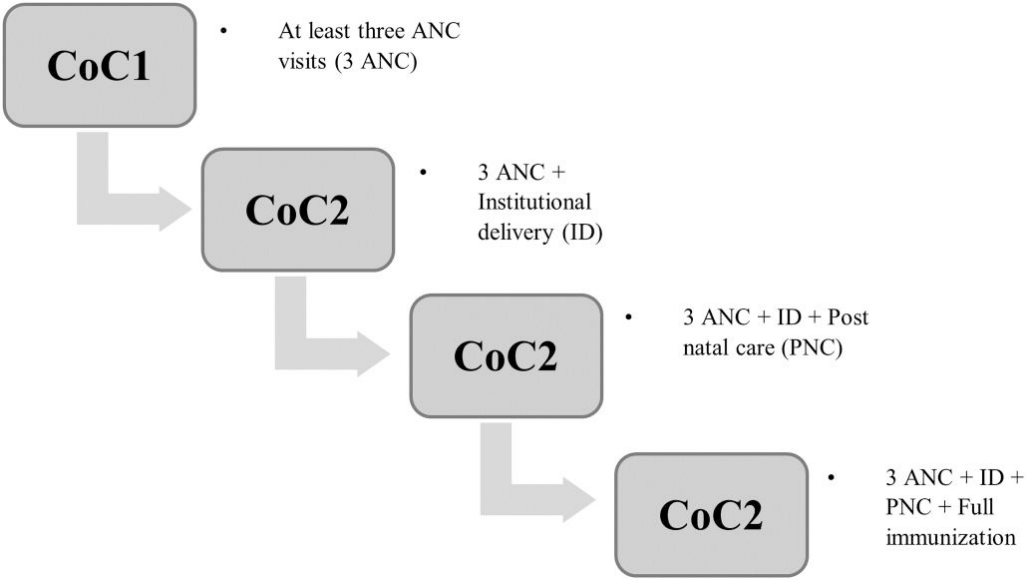
Definition of the continuum of maternal and child health care.

The CoC in MCH services is the primary outcome variable, capturing integrated service delivery for mothers and children from prepregnancy through delivery, the immediate postnatal period, and early childhood. CoC has two dimensions: (i) a time dimension—spanning prepregnancy, pregnancy, childbirth, and early childhood—and (ii) a place dimension—linking care across home, community, and health facilities (Kerber et al. 2007). Due to data limitations, our analysis focuses on the time dimension. In this study, we define CoC at different stages (Fig. 2), with each outcome measured as a binary variable coded 1 if the woman received the specific service, and 0 otherwise.

In line with the existing literature (Emiru et al. 2020, Rammohan et al. 2021, Gandhi et al. 2022), we define the four main variables of interest: CoC1 refers to women who received at least three ANC checkups. CoC2 builds on this by including institutional delivery, identifying women who accessed both ANC and delivered in a health facility. CoC3 adds PNC, capturing women who, in addition to ANC and facility-based delivery, received PNC within two months of childbirth. CoC4 incorporates child health, identifying women whose children received full immunization in addition to all earlier services. Full immunization is defined as receipt of one dose of BCG (Bacillus Calmette–Guérin), three doses each of DPT (Diphtheria, Pertussis, and Tetanus) and polio vaccines, and one dose of the measles vaccine.

To account for observed heterogeneity in program exposure and access, we control for household-level characteristics such as the sex of the household head and wealth status. At the individual level, we adjust for age, age at first birth, total number of children, years of education, rural or urban residence, religion (Hindu, Muslim, Christian, Sikh, or other), and caste group (Scheduled Tribe-ST, Scheduled Caste-SC, Other Backward Class-OBC, or Other), all of which may influence access to MCH services.

#### Methodology

***Intent-to-treat (ITT) effect:*** Our identification strategy is based on the ITT effect, employing a difference-in-difference (DiD) approach applied to a pooled cross-sectional dataset (Angrist and Krueger 1999). We leverage the program eligibility criteria outlined in the JSY guidelines to construct treatment and control groups. Although JSY was launched nationwide, eligibility rules varied across low-performing and high-performing states. We define the treatment group as women eligible for both the cash incentive and ASHA support under the program and compare their outcomes to a control group of ineligible women before and after the intervention.

We estimate the following model:


(1) $$\begin{array}{rl} Y_{iat}= & \beta_{0}+\beta_{1}Eligible_{ia}+\beta_{2}Post_{t}+\beta_{3}(Eligible_{ia}\;*\;Post_{t})\\ & +\beta_{3}X_{ia}+\alpha_{a}+\delta_{t}+\varepsilon_{iat} \end{array}$$

In line with the standard difference-in-differences specification, the model includes a dummy variable indicating whether a child was born before or after the launch of the Janani Suraksha Yojana, a dummy for program eligibility, and their interaction term capturing the program’s effect. $Y_{iat}$ is the outcome of a woman residing in state *a* who gives birth in the year *t*. $Eligible_{ia}$ is a dummy variable that takes the value 1 for the woman eligible for cash assistance in both low- and high-performing states, as defined by the program's guidelines. The variable $Post_{t}\;$ takes a value of 1 if the child is born in the postprogram launch period in our sample, i.e. year 2010 or later. The main coefficient of interest here is $\beta_{3}$, which represents the combined treatment effect of cash assistance and ASHA support for eligible mothers. $X_{ia}$ is a vector of control variables. The variable $\alpha_{a}$ denotes the state-fixed effect to control for confounding effects arising from differences in program implementation across states,^3^ and $\delta_{t}$ denotes the child's birth-year fixed effect to account for changes in access to MCH services due to advancements in medical technology or macroeconomic shocks over the years. $\varepsilon_{iat}$ is the random error term. The standard errors are clustered at the village/urban blocks level, the primary sampling unit in the NFHS.

To examine the heterogeneous effects of JSY across different subgroups of intended beneficiaries, defined by region of residence, mother’s education, and household wealth index, we estimate a triple-difference specification by interacting the Eligible*Post term with key individual and household characteristics. The estimating equation is as follows:


(2) $$\begin{array}{rl} Y_{iat} & =\;\beta_{0}+\;\beta_{1}(Eligible_{ia})+\;\beta_{2}Post_{t}+\;\beta_{3}(Characteristics_{ia})\\ & \;+\beta_{4}(Eligible_{ia}\;*\;Post_{t})+\beta_{5}(Eligible_{ia\;}*\;Characteristics_{ia})\\ & \;+\;\beta_{6}(Post_{t}\;*\;Characteristics_{ia})\\ & \;+\;\beta_{7}(Eligible_{ia}\;*\;Post_{t}\;*\;Characteristics_{ia})\;+\;X_{iat}\\ & \;+\;\alpha_{a}\;+\;\delta_{t}+\varepsilon_{iat} \end{array}$$

Here, the coefficient of interest is $\beta_{7}$, which represents the effect of the triple interaction term. The term $Characteristics_{ia}$ denotes a dummy variable indicating whether the woman is uneducated, poor, or belongs to a rural region. All other model specifications remain the same as in equation (1).

***Parallel trends assumption:*** The validity of the DiD approach relies on the parallel trends assumption, which states that in the absence of the program, the outcomes for the treatment and control groups would have followed similar trajectories over time. To test this, we conduct a falsification test using only preprogram data from NFHS-2 (Patwardhan 2023). Specifically, we re-estimate equation (1) using two false post-treatment indicators. The first ($False\;Post_{t}1$_)_ assumes a program starts in April 1997, and the second ($False\;Post_{t}2$_)_ assumes a start in April 1998. Each takes a value of 1 if the child was born after the respective cutoff date and 0 otherwise, while all other variables remain unchanged. If the treatment and control groups exhibited similar trends prior to the actual program rollout, the interaction coefficients should be statistically insignificant, thereby supporting the parallel trends assumption.

### Primary data for qualitative analysis

To complement the quantitative findings and explore the experiences of women and frontline CHWs with maternal and child health services—particularly the roles of ASHAs and conditional cash incentives in influencing care-seeking behavior along the CoC—we conducted qualitative interviews in 2024–2025. In August 2024, we conducted in-depth interviews with 25 mothers of infants aged 2 to 8 months from 10 randomly selected villages in the Kalyanpur block of Kanpur Nagar district, Uttar Pradesh, a socioeconomically backward Indian state. Villages were selected to capture variation in ASHA engagement, socioeconomic characteristics, and local health infrastructure. Mothers were identified through door-to-door screening, and interviews were conducted in Hindi by a trained female researcher to ensure comfort and openness. Each session lasted 15–20 minutes and followed a semi-structured, open-ended guide.

Respondents were purposively selected to represent two groups: (i) women who had completed the full CoC—comprising at least three ANC visits, institutional delivery, and PNC—and (ii) those who had accessed only part of the CoC. For both groups, we explored the perceived role of ASHAs and conditional cash incentives in enabling or hindering care continuity. Among partial-CoC cases, we first identified the specific service(s) not accessed and then probed the reasons for discontinuity, while also examining the factors that facilitated uptake of the services they did receive. Because most mothers were unfamiliar with the term *Janani Suraksha Yojana* as well as its underlying components—and because the maternity-related cash incentives and ASHA support they experienced are themselves part of JSY—we framed our interview questions around these program features rather than the program name. This allowed us to assess how program elements were experienced in practice, while also exploring contextual barriers such as sociocultural norms, financial limitations, and access to care.

In April 2025, after obtaining approval from the state health department, we conducted two group discussions with 62 ASHAs during their routine monthly cluster meetings. Discussion prompts focused on understanding why some women completed the full CoC while others did not, and elicited ASHAs’ perspectives on key facilitators and barriers to service uptake, including their own roles and the influence of financial incentives. To ensure a productive and focused dialogue and given the sensitivity of the setting, we deliberately avoided prompting discussion of broader supply-side constraints (e.g. health system staffing or incentive adequacy), which could elicit defensive or uninformative responses. Instead, the conversations centered on ASHAs’ field-level experiences in supporting maternal care and their observations on factors that enable or inhibit CoC completion.

Qualitative data were analyzed using a deductive thematic approach, guided by the study’s conceptual framework, and informed by insights from the quantitative analysis. Transcripts were systematically coded and organized into themes and subthemes that captured patterns in care access as well as the contextual factors that enabled, constrained, or shaped women’s engagement with the CoC.

## Results

### Quantitative results

#### Summary statistics and descriptive results

In our study sample, the average age of women was 26.22 years, and they had an average of 2.36 children (Table 1). The average age at first birth increased from 19.46 years in the preprogram period to 21.33 years after the program launch. Women had completed an average of 6.03 years of education, increasing from 3.9 years in NFHS-2 to 6.47 years in NFHS-4. Around 77% of the sample resided in rural areas. In terms of caste distribution, 19% belonged to the Scheduled Tribes (ST), 19% to the Scheduled Castes (SC), and 38% to the Other Backwards Classes (OBC). Regarding religion, 69% of the women identified as Hindu, 17% as Muslim, and 9% as Christian.

**Table 1:** Summary statistics of key variables.

Variable	NFHS-2		NFHS-4		Overall	
	Mean	Std. Dev.	Mean	Std. Dev.	Mean	Std. Dev.
***Individual characteristics***						
Age	25.38	5.15	26.4	5.01	26.22	5.05
Age at first birth	19.46	3.5	21.33	3.71	21.01	3.74
Children ever born	2.71	1.63	2.28	1.36	2.36	1.42
Years of education	3.90	4.77	6.47	5.21	6.03	5.23
**Rural-urban**						
Urban	0.24	0.43	0.23	0.42	0.23	0.42
Rural	0.76	0.43	0.77	0.42	0.77	0.42
**Caste**						
ST	0.18	0.39	0.19	0.40	0.19	0.39
SC	0.14	0.35	0.21	0.40	0.19	0.40
OBC	0.25	0.44	0.41	0.49	0.38	0.49
Others	0.42	0.49	0.19	0.40	0.23	0.42
**Religion**						
Hindu	0.71	0.45	0.68	0.47	0.69	0.46
Muslim	0.16	0.37	0.18	0.38	0.17	0.38
Christian	0.08	0.27	0.09	0.29	0.09	0.29
Sikh	0.03	0.16	0.02	0.14	0.02	0.15
Others	0.02	0.16	0.03	0.16	0.03	0.16
***Household characteristics***						
**Wealth**						
Poorest	0.18	0.38	0.25	0.43	0.24	0.43
Poorer	0.19	0.39	0.24	0.43	0.23	0.42
Middle	0.21	0.40	0.20	0.40	0.20	0.40
Richer	0.23	0.42	0.17	0.37	0.18	0.38
Richest	0.19	0.40	0.15	0.35	0.15	0.36
**Household head**						
Male	0.93	0.26	0.87	0.34	0.88	0.33
Female	0.07	0.26	0.13	0.34	0.12	0.33
***Outcome variables***						
At least 1 ANC	0.45	0.50	0.61	0.49	0.58	0.49
Facility delivery	0.34	0.47	0.77	0.42	0.69	0.46
Postnatal checkup	0.31	0.46	0.54	0.50	0.49	0.50
Full immunization	0.27	0.44	0.43	0.50	0.40	0.49
CoC1	0.45	0.50	0.61	0.49	0.58	0.49
CoC2	0.26	0.44	0.52	0.50	0.48	0.50
CoC3	0.17	0.38	0.29	0.45	0.26	0.44
CoC4	0.08	0.27	0.13	0.33	0.12	0.32
**Observations (N)**	**21 603**		**104 852**		**126 455**	

Notes: The table presents weighted sample means and standard deviations for the baseline and endline periods. CoC1–CoC4 are defined as follows: CoC1 – women with uptake of at least three antenatal care (ANC) check–ups; CoC2 – CoC1 plus institutional delivery; CoC3 – CoC2 plus postnatal care (PNC) within two months of childbirth; and CoC4 – CoC3 plus the child received full immunization (one dose of BCG, three doses each of DPT and polio vaccines, and one dose of measles vaccine). The statistics are calculated using data from NFHS–2 and NFHS–4. The bold values in the “Observations” row indicate the unweighted sample size of women in NFHS–2 (21 603), NFHS–4 (104 852), and the pooled sample across both rounds (126 455).

The second panel of the table presents household characteristics. About 88% of households had a male head, and nearly 24% were in the poorest wealth quintile. We observed improvements in all CoC outcome indicators between the pre and postprogram launch periods. The percentage of women who completed CoC1, CoC2, CoC3, and CoC4 increased from 45% to 61%, from 26% to 52%, from 17% to 29%, and from 8% to 13%, respectively.

**Figure 3:**
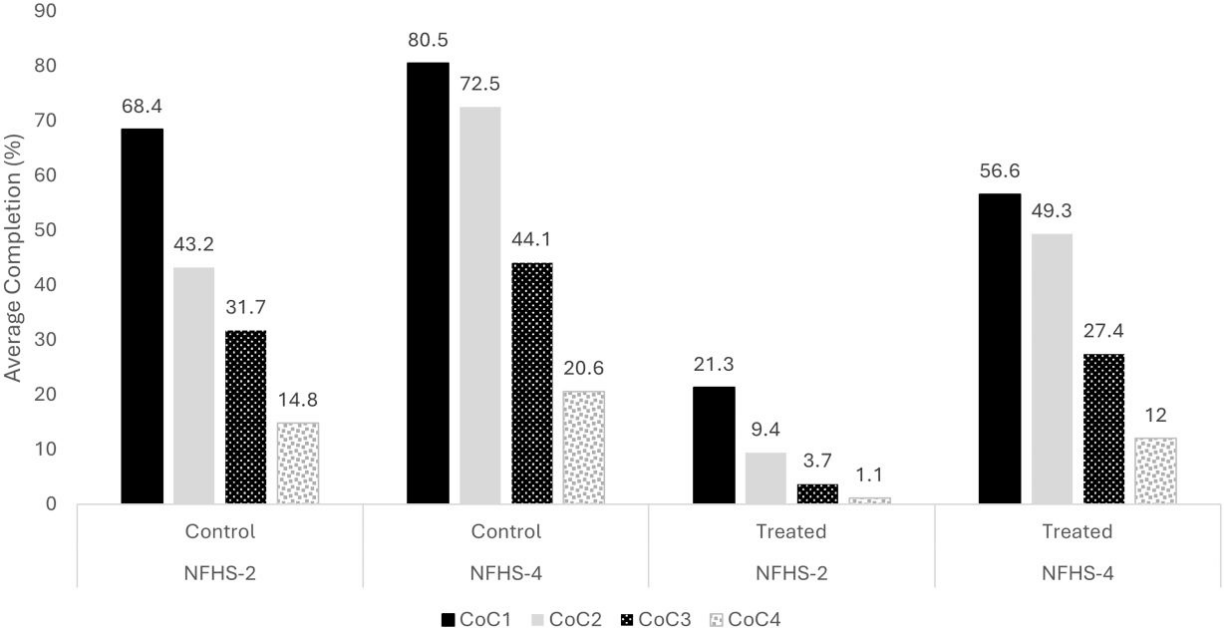
Access to the continuum of maternal and child health care among treated and control women, pre and postprogram launch.

Figure 3 illustrates the percentage of JSY-eligible and ineligible women who accessed the CoC across the pre and postprogram launch periods. While both the treated and control groups showed dropouts at later stages of the CoC, service uptake increased at each stage across the two rounds. Among JSY-eligible women, the proportion receiving at least three ANC visits increased significantly from 21.3% to 56.6%, whereas the rise for the control group was from 68.4% to 80.5%. Uptake in the second and third stages of the CoC more than tripled for the treated group, increasing from 9.4% to 49.3% and from 3.7% to 27.4%, respectively. Between the two rounds, the share of treated women who completed the full continuum of services increased from 1.1% to 12%, whereas the corresponding figure for the control group rose from 14.8% to 20.6%. The figure clearly shows that JSY-eligible women experienced greater improvement in CoC uptake between the two rounds than their ineligible counterparts.

A comparison of access to CoC among rural and urban women (Supplementary Figure A1) shows a significant increase in both areas between the two rounds. The rate of improvement was higher among women in the treated group in both regions. However, the increase was larger among urban eligibles, with the retention rate through the final stage of CoC rising from 3.8% to 16.8% in the postprogram launch period, compared with an increase from 0.7% to 10.9% among rural eligibles. A caste-wise analysis (Supplementary Figure A2) reveals that, following the program’s implementation, JSY-eligible women across all caste groups experienced a higher percentage increase in CoC uptake compared to ineligible women. However, the improvement was more pronounced among women from low-caste (SC/ST) backgrounds than among those from upper-caste backgrounds.

#### ITT estimates

In Table 2, columns (1)–(8) present the effect of JSY and ASHA on CoC uptake in MCH services. The coefficient on *Eligible* reflects the baseline difference in CoC completion before JSY, indicating that eligible women had a lower initial probability of completing the CoC than ineligible women. The *Post* coefficient captures the overall time trend common to both groups. For CoC1–CoC3, this coefficient was positive, indicating a secular trend of improvements in service uptake over time. In contrast, for CoC4, the *Post* coefficient is negative, indicating that the completion of the full continuum of care declined over time for both groups, regardless of JSY. Across all CoC measures, the positive and statistically significant interaction terms indicates that eligible women experienced a larger improvement in CoC completion relative to ineligible women after JSY was introduced. Column (1) further shows that JSY increased the probability of receiving at least three ANC visits by 16 percentage points (*P* < 0.01), and this estimate remained robust—slightly reduced to 12.1 percentage points—in Column (2) after adjusting for individual and household covariates.

**Table 2:** Effect of JSY on the continuum of maternal and child health care.

	CoC1		CoC2		CoC3		CoC4	
	(1)	(2)	(3)	(4)	(5)	(6)	(7)	(8)
Eligible	−0.126***	−0.114***	−0.019	−0.002	−0.039***	−0.025***	−0.043***	−0.039***
	(0.012)	(0.009)	(0.013)	(0.009)	(0.011)	(0.008)	(0.007)	(0.006)
Post	0.223***	0.209***	0.357***	0.336***	0.216***	0.216***	−0.115***	−0.113***
	(0.014)	(0.011)	(0.015)	(0.011)	(0.013)	(0.011)	(0.009)	(0.008)
Eligible × post	0.164***	0.121***	0.076***	0.027***	0.089***	0.050***	0.063***	0.046***
	(0.012)	(0.009)	(0.013)	(0.009)	(0.010)	(0.008)	(0.007)	(0.006)
Controls	No	Yes	No	Yes	No	Yes	No	Yes
State FE	Yes	Yes	Yes	Yes	Yes	Yes	Yes	Yes
Birth year FE	Yes	Yes	Yes	Yes	Yes	Yes	Yes	Yes
Observations	125 491	119 708	125 706	119 914	102 428	98 487	113 644	108 910
*R*-squared	0.170	0.253	0.178	0.293	0.141	0.217	0.090	0.124

*Notes*: The table presents intent-to-treat (ITT) estimates. Columns (1)–(8) report results for four successive stages of the maternal and child health (MCH) continuum of care (CoC1–CoC4) as dependent variables. CoC1–CoC4 are defined as follows: CoC1—uptake of at least three antenatal care (ANC) check-ups; CoC2–CoC1 plus institutional delivery; CoC3–CoC2 plus postnatal care (PNC) within two months of childbirth; and CoC4–CoC3 plus the child received full immunization (one dose of BCG, three doses each of DPT and polio vaccines, and one dose of measles vaccine). *Eligible* denotes women eligible under the program. *Post* is a binary indicator equal to 1 for births occurring in the postprogram launch period. The models control for individual characteristics, including the mother’s age, age at first birth, number of children born, years of education, caste, religion, and residential location (rural/urban), as well as household characteristics such as household wealth and sex of the household head. They also include the child’s year of birth and state fixed effects. Standard errors are clustered at the PSU level and reported in parentheses. *** *P* < 0.01, ** *P* < 0.05, * *P* < 0.1. Source: Authors’ calculations using NFHS-2 and NFHS-4.

Columns (3) and (4) show the impact on combined ANC and institutional delivery. The interaction term Eligible*Post indicates that program eligibility increased the likelihood of receiving both services by 7.6 percentage points (*P* < 0.01) in the unadjusted model and by 2.7 percentage points (*P* < 0.01) when covariates are included. Column (5) reveals that the likelihood of receiving ANC, institutional delivery, and PNC in sequence increased by 8.9 percentage points (*P* < 0.01) for eligible women. This effect remained strong at 5 percentage points (*P* < 0.01) after including controls (Column 6).

Finally, Columns (7) and (8) present estimates for the most comprehensive measure, CoC4, which includes full child immunization. The results show that eligible women were 4.6 percentage points (*P* < 0.01) more likely to complete the full continuum of MCH services, even after adjusting for controls. To further illustrate the estimated program effects, we computed postestimation predicted probabilities for each outcome by eligibility and period and graphed them with 95% confidence intervals. The trends in Supplementary Figure A3 align with the regression estimates: eligible women started at lower CoC uptake than non-eligible women but exhibited a larger postprogram increase.

#### Heterogeneity analysis

The heterogeneous effects for women across regions of residence are presented in Table 3. Column (1) shows a negative but statistically insignificant coefficient for rural women. However, columns (2)–(4) report negative and statistically significant estimates, indicating that JSY-eligible women in rural areas did not experience a disproportionately greater increase in access to the continuum of MCH services compared to their urban counterparts. In fact, urban eligible women had more than 20 percentage points higher uptake of the continuum of ANC, institutional delivery, and PNC services. This suggests that while the program improved service access across both rural and urban areas, the increase was more substantial among urban women.

**Table 3:** Heterogeneous effects of JSY on the continuum of maternal and child health care by rural and urban residence.

	CoC1	CoC2	CoC3	CoC4
	(1)	(2)	(3)	(4)
Eligible	−0.099***	−0.082***	−0.147***	−0.102***
	(0.017)	(0.019)	(0.017)	(0.012)
Post	0.129***	0.221***	0.111***	−0.177***
	(0.012)	(0.015)	(0.016)	(0.012)
Rural	−0.127***	−0.207***	−0.133***	−0.072***
	(0.010)	(0.013)	(0.013)	(0.010)
Eligible × post	0.125***	0.107***	0.197***	0.128***
	(0.018)	(0.020)	(0.018)	(0.013)
Eligible × rural	−0.001	0.125***	0.167***	0.087***
	(0.019)	(0.019)	(0.016)	(0.012)
Post × rural	0.121***	0.176***	0.156***	0.096***
	(0.013)	(0.015)	(0.017)	(0.012)
Eligible × post ×rural	−0.030	−0.129***	−0.203***	−0.115***
	(0.021)	(0.021)	(0.020)	(0.014)
Controls	Yes	Yes	Yes	Yes
State FE	Yes	Yes	Yes	Yes
Birth year FE	Yes	Yes	Yes	Yes
Observations	119 708	119 914	98 487	108 910
*R*-squared	0.254	0.295	0.219	0.125

*Notes*: The table presents estimates of *β*_1_, *β*_4_, and *β*_7_ from equation (2). The dummy variable *Rural* takes the value 1 for mothers residing in rural areas. CoC1–CoC4 are defined as follows: CoC1—uptake of at least three antenatal care (ANC) check-ups; CoC2–CoC1 plus institutional delivery; CoC3–CoC2 plus postnatal care (PNC) within two months of childbirth; and CoC4–CoC3 plus the child received full immunization (one dose of BCG, three doses each of DPT and polio vaccines, and one dose of measles vaccine). *Eligible* denotes women eligible under the program. *Post* is a binary variable equal to 1 for births occurring in the postprogram launch period. The models control for individual characteristics, including the mother’s age, age at first birth, number of children born, years of education, caste, and religion, as well as household characteristics such as household wealth and sex of the household head. They also include the child’s birth year and state fixed effects. Standard errors are clustered at the PSU level and reported in parentheses. *** *P* < 0.01, ** *P* < 0.05, * *P* < 0.1. Source: Authors’ calculations using NFHS-2 and NFHS-4.

Table 4 presents the heterogeneity of the program impact by mother’s education. The coefficients on the triple interaction terms are negative and statistically significant across all outcome variables, indicating that the program led to a greater increase in access to the CoC of MCH services among educated women than among uneducated women.

**Table 4:** Heterogeneous effects of JSY on the continuum of maternal and child health care by maternal education (illiterate vs. literate).

	CoC1	CoC2	CoC3	CoC4
	(1)	(2)	(3)	(4)
Eligible	−0.129***	−0.090***	−0.117***	−0.087***
	(0.012)	(0.012)	(0.011)	(0.008)
Post	0.186***	0.318***	0.205***	−0.125***
	(0.011)	(0.012)	(0.012)	(0.009)
Illiterate	−0.192***	−0.212***	−0.143***	−0.080***
	(0.010)	(0.010)	(0.010)	(0.008)
Eligible × post	0.136***	0.113***	0.141***	0.096***
	(0.012)	(0.012)	(0.011)	(0.008)
Eligible × illiterate	0.078***	0.192***	0.177***	0.097***
	(0.014)	(0.013)	(0.012)	(0.008)
Post × illiterate	0.095***	0.095***	0.065***	0.053***
	(0.013)	(0.013)	(0.013)	(0.010)
Eligible × post × illiterate	−0.083***	−0.185***	−0.172***	−0.106***
	(0.017)	(0.016)	(0.015)	(0.010)
Controls	Yes	Yes	Yes	Yes
Birth year FE	Yes	Yes	Yes	Yes
State FE	Yes	Yes	Yes	Yes
Observations	119 721	119 927	98 500	108 923
*R*-squared	0.247	0.282	0.111	0.091

*Notes*: The table presents estimates of *β*_1_, *β*_4_, and *β*_7_ from equation (2). The dummy variable *Illiterate* takes the value 1 for mothers with no formal education. CoC1–CoC4 are defined as follows: CoC1—uptake of at least three antenatal care (ANC) check-ups; CoC2–CoC1 plus institutional delivery; CoC3–CoC2 plus postnatal care (PNC) within two months of childbirth; and CoC4–CoC3 plus the child received full immunization (one dose of BCG, three doses each of DPT and polio vaccines, and one dose of measles vaccine). *Eligible* denotes women eligible under the program. *Post* is a binary variable equal to 1 for births occurring in the postprogram launch period. The models control for individual characteristics, including the mother’s age, age at first birth, number of children born, caste, religion, and residential location (rural/urban), as well as household characteristics such as household wealth and sex of the household head. They also include the child’s birth year and state fixed effects. Standard errors are clustered at the PSU level and reported in parentheses. *** *P* < 0.01, ** *P* < 0.05, * *P* < 0.1. Source: Authors’ calculations using NFHS-2 and NFHS-4.

Table 5 reports heterogeneity by household wealth. The coefficients of interest are consistently negative and statistically significant for women in the bottom two wealth quintiles, indicating that those from the top three quintiles experienced significantly greater gains in accessing a continuum of MCH services.

**Table 5:** Heterogeneous effects of JSY on the continuum of maternal and child health care by wealth status (poor vs. non-poor).

	CoC1	CoC2	CoC3	CoC4
	(1)	(2)	(3)	(4)
Eligible	−0.119***	−0.070***	−0.102***	−0.077***
	(0.012)	(0.012)	(0.011)	(0.007)
Post	0.176***	0.320***	0.198***	−0.122***
	(0.011)	(0.012)	(0.012)	(0.009)
Poor	−0.183***	−0.196***	−0.134***	−0.062***
	(0.013)	(0.013)	(0.012)	(0.008)
Eligible × post	0.128***	0.076***	0.119***	0.081***
	(0.012)	(0.012)	(0.012)	(0.008)
Eligible × poor	0.042**	0.154***	0.153***	0.077***
	(0.016)	(0.015)	(0.014)	(0.008)
Post × poor	0.092***	0.042***	0.048***	0.024**
	(0.015)	(0.014)	(0.015)	(0.010)
Eligible × post × poor	−0.064***	−0.120***	−0.157***	−0.080***
	(0.018)	(0.017)	(0.016)	(0.010)
Controls	Yes	Yes	Yes	Yes
Birth year FE	Yes	Yes	Yes	Yes
State FE	Yes	Yes	Yes	Yes
Observations	119 708	119 914	98 487	108 910
*R*-squared	0.248	0.288	0.213	0.122

*Notes*: The table presents estimates of *β*_1_, *β*_4_, and *β*_7_ from equation (2). The variable *Poor* takes the value 1 for women in the bottom two quintiles of the wealth index (poorest and poorer categories). CoC1–CoC4 are defined as follows: CoC1—uptake of at least three antenatal care (ANC) check-ups; CoC2–CoC1 plus institutional delivery; CoC3–CoC2 plus postnatal care (PNC) within two months of childbirth; and CoC4–CoC3 plus the child received full immunization (one dose of BCG, three doses each of DPT and polio vaccines, and one dose of measles vaccine). *Eligible* denotes women eligible under the program. *Post* is a binary variable equal to 1 for births occurring in the postprogram launch period. The models control for individual characteristics, including the mother’s age, age at first birth, number of children born, years of education, caste, religion, and residential location (rural/urban), as well as household characteristics such as household wealth and sex of the household head. They also include the child’s birth year and state fixed effects. Standard errors are clustered at the PSU level and reported in parentheses. *** *P* < 0.01, ** *P* < 0.05, * *P* < 0.1. Source: Authors’ calculations using NFHS-2 and NFHS-4.

Together, these findings suggest that among the socioeconomically disadvantaged groups targeted by the JSY, the benefits of accessing a continuum of MCH services were not uniformly distributed. Within this population, women who were relatively better off—those residing in urban areas, having some formal education, or belonging to higher wealth quintiles—experienced significantly greater improvements in the uptake of a continuum of services compared to their rural, less educated, and poorer counterparts. This indicates that, although the program successfully reached disadvantaged populations, structural and socioeconomic differences within this group influenced the extent to which women could benefit from the full continuum of MCH services.

#### Falsification test

Supplementary Table A1 presents the results of the falsification test. Columns (1) through (8) report the coefficients of the interaction term estimated using only preprogram data. For CoC1 to CoC3, the estimates are small and statistically insignificant, indicating that the treatment and control groups exhibited parallel trends in access to CoC prior to the program's rollout. However, the coefficient for CoC4 is statistically significant, indicating a potential pre-existing trend in full continuum completion. This raised concerns about the robustness of the causal interpretation of the CoC4 estimates. Thus, across CoC1 to CoC3, our results satisfy the parallel trends assumption, whereas those for CoC4 should be interpreted with caution.

#### Additional results and robustness tests

Beyond the main estimates using CoC as the outcome, we assess the impact of JSY on each constituent stage of MCH services. Supplementary Table A2 presents these disaggregated effects. Columns (1) and (2) showed that program eligibility increased the likelihood of receiving at least one ANC check-up by 24.4 percentage points and institutional delivery by 17.5 percentage points. Columns (3) and (4) indicate a rise in PNC and full immunization by 17.1 and 21.9 percentage points, respectively, among eligible women compared to non-eligible counterparts. These results reinforce that the program enhanced access to individual maternal care services and to the full continuum. However, the aggregate CoC effect was slightly lower than for individual stages.

We assessed heterogeneity in program effects across state performance categories. The treatment groups comprise eligible women from both low-performing and high-performing states, while the control groups in each case consist of non-eligible women from high-performing states. As shown in Supplementary Tables A3 and A4, JSY significantly improved access to MCH services across both groups. Notably, the effects were stronger in low-performing states for CoC1 and CoC4, whereas for outcomes CoC2 and CoC3, the estimated coefficient was larger in high-performing states. These findings support the robustness and generalizability of our main results.

As a robustness check, we re-estimate equation (1) using a matched sample constructed via propensity score matching (PSM). Propensity scores are estimated using a probit model with covariates that include maternal age, age at first birth, parity, education, caste, religion, residence (urban/rural), household wealth, and the sex of the household head. Treated (eligible) women are matched with five non-eligible women using nearest-neighbor matching (Himaz 2008). Results presented in Supplementary Table A5 closely mirror the main findings, confirming a significant positive impact of JSY on CoC uptake in the matched sample. As a robustness check to address the long interval between the pre and postprogram launch periods, we re-estimated the models using NFHS-3 (2005–2006) as the preprogram period. Because NFHS-3 lacked postnatal care information, we defined an alternative continuum-of-care outcome (New_CoC3) as completion of ANC, institutional delivery, and child immunization. To maintain comparability, both rounds were restricted to births within the three years preceding the survey. We estimated the same specification, including *eligibility*, *Post,* and their interaction, with standard covariates and fixed effects. The results (Supplementary Table A6) were positive and statistically significant, consistent with the main findings. Lastly, as a robustness check for the parallel trend assumption, we plotted CoC outcomes for eligible and non-eligible women across NFHS-2, NFHS-3, and NFHS-4, harmonizing the reference period to births within three years prior to each survey. As shown in Supplementary Figure A4, trends for eligible and non-eligible groups were broadly similar for CoC2 and New_CoC3, consistent with parallel trends. For CoC1, the eligible group exhibited a slightly steeper preprogram increase; therefore, estimates for this outcome should be interpreted with appropriate caution.

### Findings from qualitative data

Among the 25 mothers interviewed, 13 reported receiving the full CoC, including at least three ANC checkups, institutional delivery, and PNC. Of these, eight were delivered in public health facilities and five in private hospitals. However, only three women reported receiving the JSY cash transfer. The findings are presented below in two groups: women who received the complete CoC and those who accessed only a partial CoC.

#### Women with a complete continuum of care

Among those who completed the full CoC, the consistent engagement of ASHAs emerged as the most significant factor enabling their success. Respondents described ASHAs providing both logistical and emotional support—assisting with pregnancy registration, distributing iron-folic acid (IFA) supplements, arranging transportation, and facilitating newborn immunizations. However, variation in ASHA engagement was evident across locations.

‘ASHA came regularly to our home during my pregnancy. She would check on my health every 15 days as she passed by. She gave me iron tablets and came every time I needed help, especially during emergencies. She is very good.’ (Participant who completed full CoC)

‘ASHA came once or twice to meet me and gave me iron tablets. I had already registered my pregnancy at a private hospital. Once she found out that I was going for checkups there, she stopped visiting.’ (Participant who completed full CoC)

In group discussions, ASHAs acknowledged the importance of financial incentives, such as JSY, but emphasized that the amount was insufficient to motivate women to complete the full continuum independently.

#### Women with partial continuum of care

In-depth interviews with mothers and group discussions with ASHAs revealed that several contextual barriers disrupted women’s progression through the full maternal care continuum. These discontinuities occurred at various stages—some women did not complete the recommended ANC visits, others opted for home delivery, and several failed to obtain PNC. The following themes emerged as critical determinants of partial uptake.

***Social-cultural norms*:** ASHAs highlighted that in rural areas, some women conceal early pregnancies due to cultural beliefs or fear of miscarriage, which delays or prevents early ANC visits. While institutional deliveries are increasingly accepted, resistance remains in some of the households, particularly among older family members, who view home births as safe and culturally preferable. Women living in joint families often lacked autonomy over healthcare decisions.

‘Her mother-in-law said that in our family, we do not go to the hospital. They had also delivered at home, and there were no issues at that time.’ (ASHA, Kalyanpur)

‘Some women in villages keep their pregnancy a secret, especially those who have had a miscarriage in the past. They go to the hospital only in the later stages of pregnancy.’ (ASHA, Kalyanpur)

***Transport constraints:*** In several cases, home deliveries occurred due to an inability to reach a health facility on time. Transportation delays, particularly during emergencies or preterm labor, were a recurring concern.

‘I used to go to the hospital for regular checkups. On the day of delivery, I was fasting and suddenly started having labor pains. By the time I decided to go to the hospital, I had already delivered the baby.’ (Participant with partial CoC—Home Delivery)

‘The mother called me to arrange an ambulance as she started having pain before her due date. I arranged for the ambulance, but the baby was delivered at home.’ (ASHA, Kalyanpur)

***Perceived quality of care at public health facilities:*** Poor infrastructure and inadequate staffing in public hospitals emerged as key deterrents to institutional deliveries. Recurrent referrals to private facilities during complications reduced trust in government hospitals. Several women and ASHAs reported prior negative experiences with service delivery, making it difficult for ASHAs to convince families to utilize public healthcare services.

‘People do not want to go to public hospitals nowadays. Those who can afford it go to private facilities. Others ask us to take responsibility for any complications if we take them to a public hospital. So, we do not force them.’ (ASHA, Kalyanpur)

***Limited effectiveness of cash incentives:*** The amount of the cash incentive is often less than the total expenses of facility-based childbirth, which is one reason for home delivery. Many participants noted that the costs of frequent hospital visits, medicines purchased outside, and informal payments often outweighed the cash benefits received under JSY. Sometimes, mothers do not have bank accounts or supporting documents to avail of JSY benefits. The majority of JSY applicants face delays in receiving payments. ASHA also reported that the amount of cash incentives they receive for assisting pregnant women is very low (there have been no significant revisions to the cash incentive amount since its inception).

‘ASHA comes regularly, and I am also aware of the JSY scheme, but the amount of the incentive is not enough to cover the total expenses of delivery. We are very poor, and my mother is a Dai (traditional birth attendant), so I delivered my baby under her guidance.’ (Participant with partial CoC—Home Delivery)

‘JSY is not that effective in influencing people in today's time. The cash benefit helps, but it is not sufficient to cover the total hospital expenses as there are some (incidental) expenses at public hospitals.’ (ASHA, Kalyanpur)

***Limited awareness of postnatal care (PNC):*** Postnatal services were the most frequently omitted component of the continuum. Both mothers and ASHAs reported that women typically sought postnatal care only if complications arose. The absence of perceived health issues often led to skipped PNC visits, reflecting low awareness of their preventive importance.

‘I delivered my baby in the public hospital. It was a normal delivery. After 24 hours, I was discharged, and after that, I did not go back because we were both fine. I would have gone if there had been any issues. Otherwise, it is not necessary.’ (Participant with partial CoC—No PNC)

‘People do not want to go to the hospital after getting discharged. Even if we ask them to go for checkups, they say they will go if they have some issue’. (ASHA, Kalyanpur)

Overall, the partial uptake of MCH services was shaped by a combination of demand-side and supply-side barriers, including restrictive gender norms, transportation challenges, concerns about the quality of public care, insufficient financial incentives, and limited awareness of the full range of services. These constraints were most acute among women from socioeconomically disadvantaged backgrounds—the intended beneficiaries of the JSY program. While awareness and utilization of ANC and institutional delivery are gradually improving, PNC remains underutilized. Notably, qualitative findings suggest that sustained engagement by ASHAs played a more influential role in enabling CoC uptake than financial incentives alone, highlighting the centrality of trusted community-level support in overcoming structural barriers to care.

## Discussion

The study findings indicate that JSY not only improved individual service components but also strengthened continuity across antenatal, delivery, and postnatal stages—particularly when financial incentives were combined with ASHA engagement. This integrated impact, evident in both quantitative ITT estimates and qualitative insights, underscored the effectiveness of coupling demand- and supply-side interventions. While previous studies have shown that JSY improved MCH utilization, our contribution lies in examining service use as a continuum rather than as isolated events, and in emphasizing the role of sustained CHW engagement in promoting holistic care.

Qualitative analysis conducted nearly a decade after the NFHS-4 data reinforces the program's sustained impact and institutionalization, demonstrating how its integrated design has continued to influence the uptake of MCH services over time. The qualitative interviews suggest that mothers receiving both the cash incentive and ASHA support, or ASHA support alone, were more likely to complete the CoC than those receiving only the financial incentive. A plausible explanation is that ASHAs’ incentives are explicitly tied to contacts across antenatal, intrapartum, and postnatal stages, reinforcing continuity of care. These insights are consistent with emerging quantitative evidence that combining JSY cash assistance with ASHA support improves short-term birth outcomes (Baulia 2023) and corroborate earlier findings highlighting ASHAs’ role in increasing MCH service uptake (Vellakkal et al. 2017, Agarwal et al. 2019). However, distinguishing the relative causal contribution of ASHA support versus cash transfers requires further quantitative research that exploits direct variation in the intensity and design of community health workers and the cash transfer.

Nonetheless, the study highlights persistent contextual barriers to completing the full CoC. Consistent with existing literature, limited family support, financial hardship, and sociocultural norms continue to hinder access to the recommended minimum number of ANC visits (Agarwal et al. 2019). Additionally, some women discontinue care due to perceived poor service quality, long wait times, or unwelcoming facility environments (Konje et al. 2018). Moreover, because JSY incentives for mothers are primarily tied to institutional delivery, rather than the full CoC, dropouts along the care pathway remain common. Even nearly two decades after JSY’s introduction, our qualitative data suggest that several of the well-documented challenges to maternal health service uptake remain prevalent. These persistent barriers underscore the importance of sustained investment in both community-level engagement and broader health system strengthening to further enhance continuity of care. Following the full implementation phase of the National Health Mission, fewer than half of the eligible low-income women accessed JSY benefits or ASHA services (Singh and Vellakkal 2023), indicating ongoing gaps in outreach.

Heterogeneity analysis further revealed that, although the program improved most CoC indicators, the gains were uneven, with substantially weaker effects among the poorest and least educated women. A key factor is the misalignment between the below-poverty-line (BPL) eligibility criteria and actual economic vulnerability, leading to the exclusion of many intended beneficiaries (Joshi and Sivaram 2014). Among these groups, limited awareness of entitlements and low exposure to health information remained critical barriers, particularly in remote settings. Although ASHAs are expected to bridge these gaps, they may have prioritized women more likely to complete care, as their incentives were tied to ANC uptake, institutional delivery, and PNC. These patterns mirror earlier findings that JSY benefits have been inequitably distributed, disproportionately bypassing the most disadvantaged (Lim et al. 2010, Joshi and Sivaram 2014).

Our findings underscore the significance of targeted public health programs, such as JSY, particularly as expanding coverage across the CoC is central to reducing maternal, newborn, and child mortality and improving overall well-being (Bryce et al. 2013, James et al. 2022). Reflecting on the existing literature and secondary data sources, and complemented by our qualitative insights, the improvements in service uptake have followed a staggered, cumulative trajectory. Initial gains in institutional delivery and skilled birth attendance were followed by increases in ANC utilization, and more gradually, improvements in PNC. These patterns highlight the catalytic role of public health interventions, such as JSY, while also underscoring the ongoing challenges in achieving full adherence to the continuum of care.

More broadly, our findings suggest several key lessons for policy and program design in low-resource settings. First, conditional cash transfers appear most effective when embedded within robust community health worker platforms. Cash alone is unlikely to sustain engagement along the continuum, whereas financial incentives combined with sustained ASHA support are associated with higher completion rates of the CoC. Qualitative evidence also suggests that ASHAs’ ongoing outreach, accompaniment, and problem-solving may, in practice, exert a stronger influence on continuity of care than the cash incentive itself, although this hypothesis warrants further quantitative testing. Second, designing incentive schedules that are explicitly linked to each stage of the CoC—for both mothers and ASHAs—can help reduce drop-offs and align frontline workers’ efforts with the goal of complete, rather than fragmented, care. Third, given that gains were concentrated among women who were relatively better off within the disadvantaged population, policy design and implementation need to prioritize more deliberately those facing layered disadvantages—rural, poorer, and less educated women—through refined eligibility criteria, stronger outreach in remote areas, and incentive structures that explicitly reward ASHAs for engaging harder-to-reach women, not only those most likely to complete care. These insights are relevant for other LMICs in Sub-Saharan Africa and South Asia, where persistent demand-side barriers and within-group inequalities continue to limit MCH service uptake. Integrated policy strategies that combine well-calibrated financial incentives with adequately trained, supported, and equity-oriented community health workers—embedded within responsive health systems—are likely to be most effective in achieving sustained and more equitable improvements in maternal and child health outcomes.

Our study has certain limitations, primarily arising from the dataset. First, CoC variables rely on self-reported data, which may be subject to recall or reporting bias. Second, we were unable to use NFHS-3—the survey closest to JSY's launch—due to substantial missing data on postnatal care. Instead, we relied on NFHS-2, which predates India's first BPL survey in 2002 and does not include BPL indicators. To approximate eligibility, we used the bottom quintile of the wealth index as a proxy for BPL. Lastly, we do not use the most recent NFHS round because a nationwide maternity benefit program—the Pradhan Mantri Matru Vandana Yojana (PMMVY), which provides ₹5000 in conditional cash transfers for the first live birth as partial wage compensation and nutritional support—was introduced in 2018. The resulting policy overlaps with JSY's eligibility in the post-2018 period, compromising identification and precluding the isolation of JSY’s causal effects.

## Conclusion

This study shows that cash incentives and ASHA engagement under JSY are associated with higher uptake of the CoC in MCH. By analyzing service use as a continuum and integrating quantitative and qualitative evidence, we highlight the central role of community health workers in translating financial incentives into completed care pathways. However, program gains were unevenly distributed: poorer, less educated, and rural women experienced smaller improvements in CoC uptake than their counterparts, underscoring the need for stronger targeting of the most disadvantaged. Qualitative interviews with mothers and ASHAs suggest that sustained ASHA engagement may have a greater influence on care continuity than cash incentives alone. Integrating demand-side transfers with supply-side outreach and service readiness may further reduce drop-offs along the CoC and help sustain improvements in maternal and child health. Overall, the findings suggest that conditional cash transfers embedded within robust community health worker platforms can catalyze improvements in the CoC in MCH, offering relevant lessons for other LMICs seeking to strengthen MCH systems.

## Supplementary Material

czag019_Supplementary_Data

## Data Availability

The quantitative data underlying this study are available from the corresponding author upon reasonable request. The qualitative data contain potentially identifiable respondent information and therefore cannot be shared; anonymised excerpts are reported in the manuscript.

## Footnotes

1 The 10 states were categorized as low-performing states based on their low institutional delivery rate. These were Assam, Bihar, Chhattisgarh, Jammu and Kashmir, Jharkhand, Madhya Pradesh, Odisha, Rajasthan, Uttar Pradesh, and Uttarakhand.

2 The names of state programs are Mukhyamantri Mazdoor Suraksha Yojana in Madhya Pradesh, Matrutva Anudan Yojana in Maharashtra, Mamata in Odisha, and Dr Muthulakshami Maternity Assistance Scheme in Tamil Nadu.

3 The program's eligibility requirements, payment procedure, and incentive amount varied from state to state. Their implementation and promotion process may lead to disparities in awareness of JSY across states (Carvalho and Rokicki 2019).

## Reflexivity statement

This study is authored by a female early-career researcher in the final stage of her PhD and a male senior researcher with long-standing engagement in health systems research and health financing in LMIC contexts. Both authors are based in a low- and middle-income country (LMIC).
