# Supplementary material for: Conditional cash incentives, community health workers, and continuum of maternal and child healthcare: evidence from India
Source: Health Policy Plan. 2026 Mar 3;41(4):612–26. doi: 10.1093/heapol/czag019 (PMC13089429; doi:10.1093/heapol/czag019)
Supplement: czag019_Supplementary_Data [file czag019_supplementary_data.docx]

**Online Appendix Materials**

**Figures**

**Figure A1.** Regional variation in the continuum of maternal and child health care among treated and control groups, pre- and post-program launch

**Figure A2.** Caste-wise utilization of continuum of maternal and child health care among treated and control groups, pre- and post-program launch

**Figure A3.** Trends in predicted probability among treated and control groups

**Figure A4.** Trends in the continuum of maternal and child health care across time among treated and control groups

**Tables**

**Table A1.** Falsification test for the parallel trends assumption

**Table A2.** Effect of JSY on maternal and child health services

**Table A3.** Impact of JSY on the continuum of maternal and child health care in low-performing states

**Table A4.** Effect of JSY on the continuum of maternal and child health care in high-performing states

**Table A5.** Effect of JSY on the continuum of care: Propensity score matching (PSM) analysis

**Table A6.** Effect of JSY on the continuum of maternal and child health care with NFHS-3 & NFHS-4

*Figure A1. Regional variation in the continuum of maternal and child health care among treated and control groups, pre- and post-program launch*


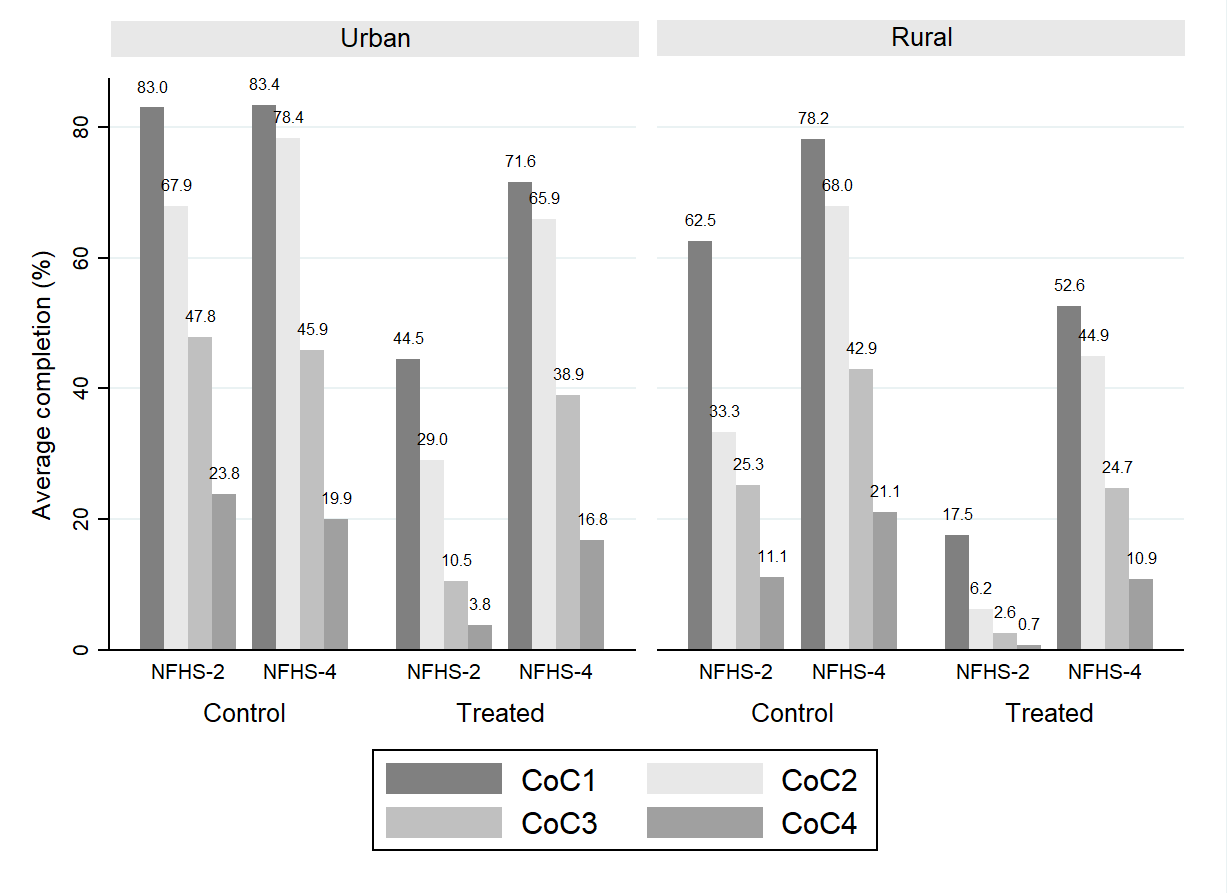


**Notes:** This figure presents utilization of the maternal continuum of care in rural and urban areas among treated and control women at baseline (NFHS-2) and endline (NFHS-4). The treated group comprises women eligible for the JSY program; women who were not eligible constitute the control group. CoC1–CoC4 are defined as follows: CoC1 – women who had uptake of at least three antenatal care (ANC) check-ups; CoC2 – CoC1 plus institutional delivery; CoC3 – CoC2 plus postnatal care (PNC) within two months of childbirth; and CoC4 – CoC3 plus the child received full immunization (one dose of BCG, three doses each of DPT and polio vaccines, and one dose of measles vaccine). Reported values are sample means. Source: Authors’ calculations using NFHS-2 and NFHS-4.

*Figure A2. Caste-wise utilization of continuum of maternal and child health care among treated and control groups, pre- and post-program launch*
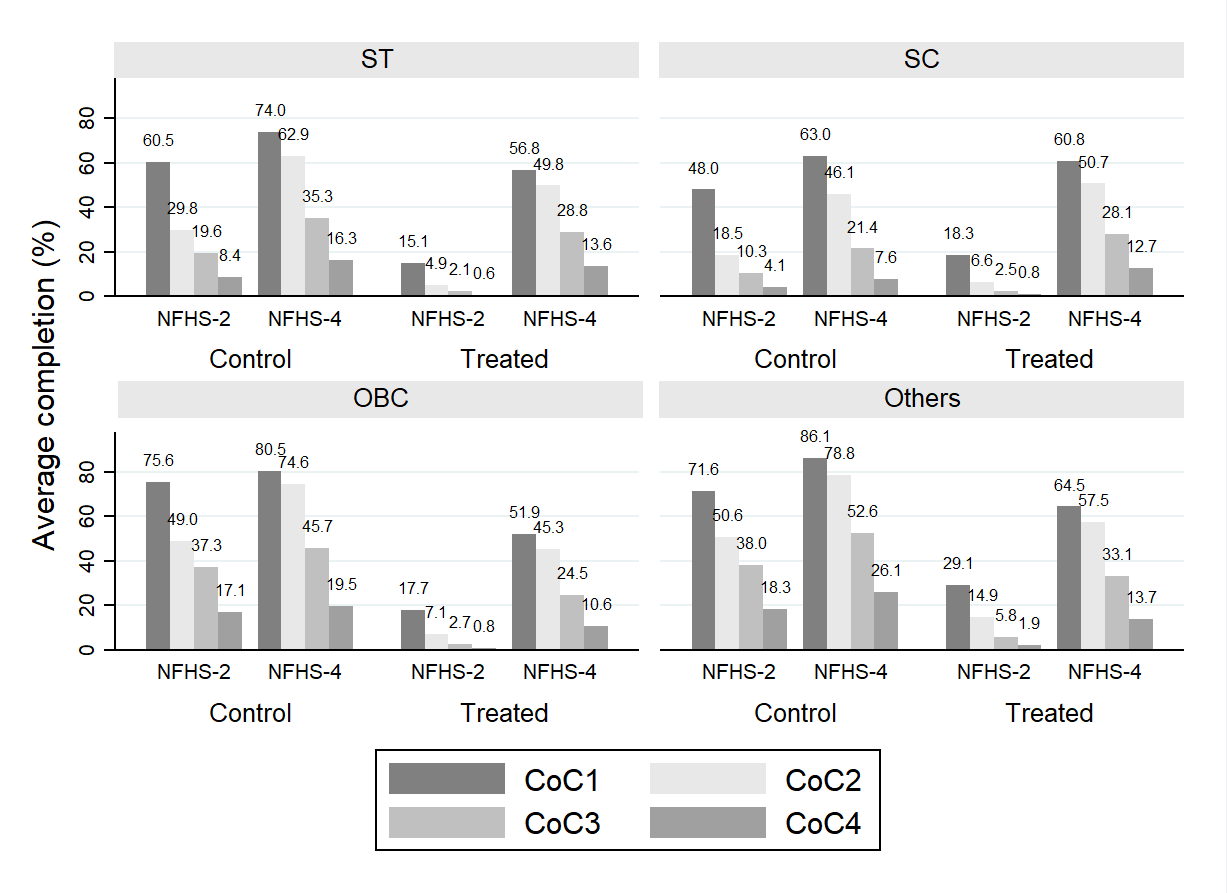


**Notes:** This figure shows caste-wise utilization of the maternal continuum of care among treated and control women at baseline (NFHS-2) and endline (NFHS-4). The treated group comprises women eligible for the JSY program; women who were not eligible constitute the control group. CoC1–CoC4 are defined as follows: CoC1 – women with uptake of at least three antenatal care (ANC) check-ups; CoC2 – CoC1 plus institutional delivery; CoC3 – CoC2 plus postnatal care (PNC) within two months of childbirth; and CoC4 – CoC3 plus the child received full immunization (one dose of BCG, three doses each of DPT and polio vaccines, and one dose of measles vaccine). Reported values are sample means. Source: Authors’ calculations using NFHS-2 and NFHS-4.

*Figure A3. Trends in predicted probability among treated and control groups*


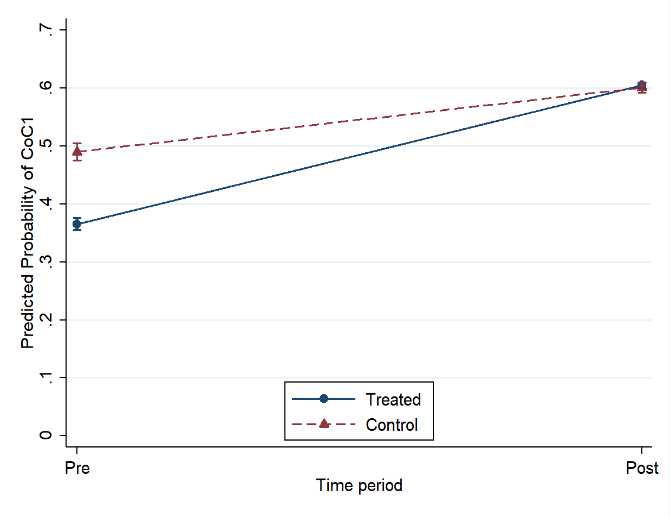

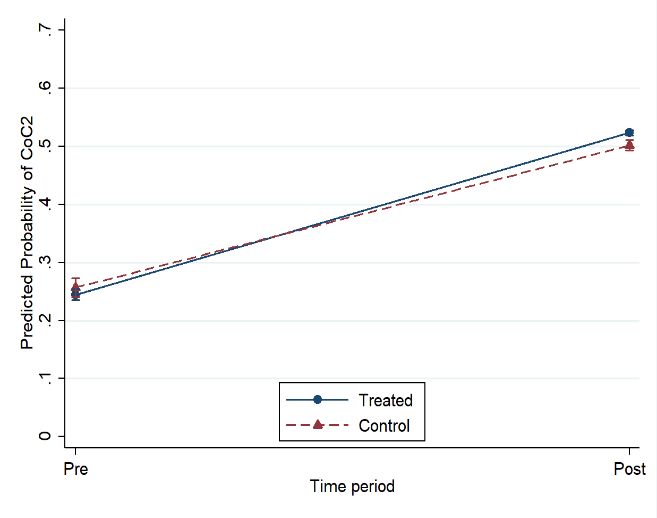


(a) (b)

*
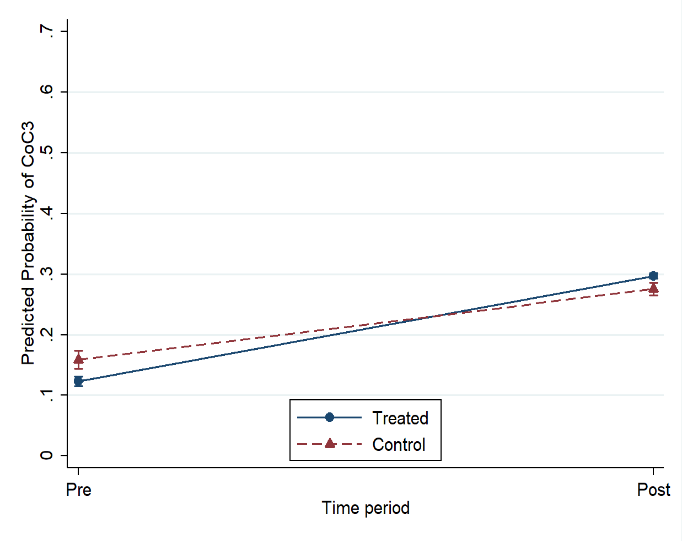

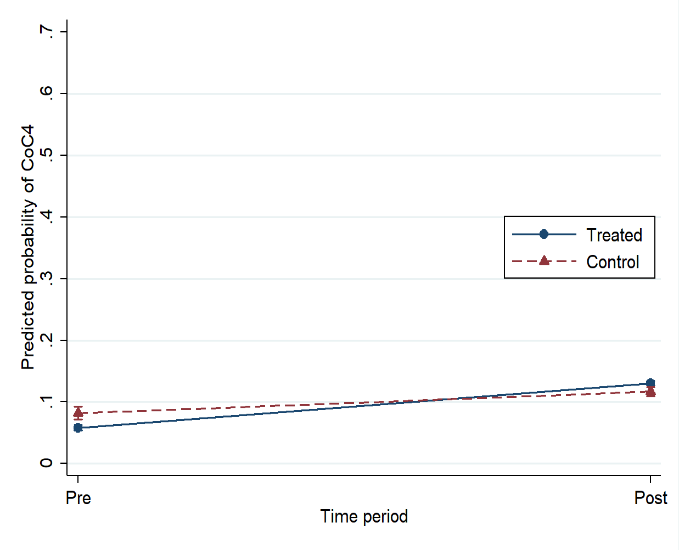
*

(c) (d)

**Notes:** This figure illustrates trends in the predicted probabilities of completing each stage of the maternal continuum of care among treated and control women. The outcome variables represent successive stages of the continuum, where a value of 1 indicates that the woman has utilized all services up to that stage. The treated group comprises women eligible for the JSY program; women who were not eligible constitute the control group. CoC1–CoC4 are defined as follows: CoC1 – uptake of at least three antenatal care (ANC) check-ups; CoC2 – CoC1 plus institutional delivery; CoC3 – CoC2 plus postnatal care (PNC) within two months of childbirth; and CoC4 – CoC3 plus the child received full immunization (one dose of BCG, three doses each of DPT and polio vaccines, and one dose of measles vaccine). The estimated model includes controls for mother’s age, age at first birth, number of children born, years of education, caste, religion, household wealth, sex of the household head, and state fixed effects. Standard errors are clustered at the PSU level. Source: Authors’ calculations using NFHS-2 and NFHS-4.

*Figure A4. Trends in the continuum of maternal and child health care across time among treated and control groups*

*
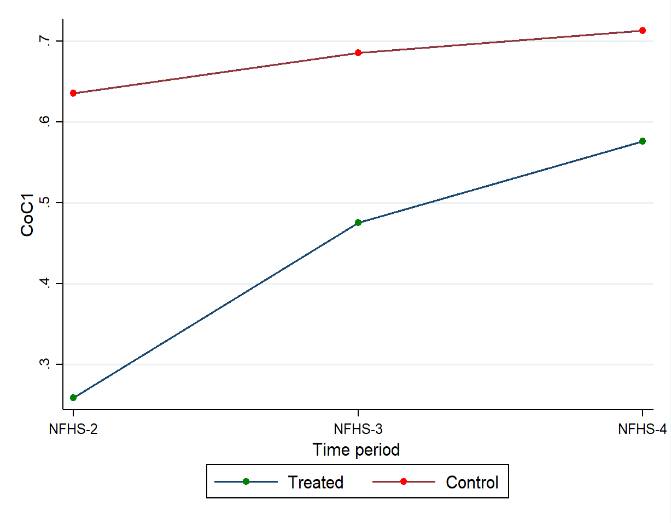

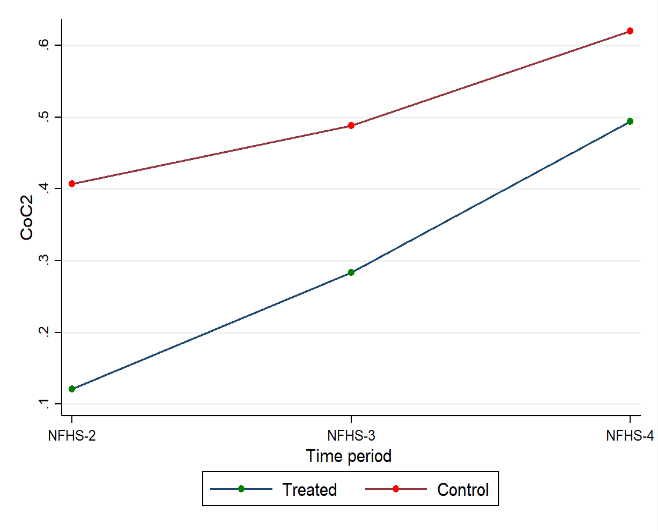
*

(a) (b)

*
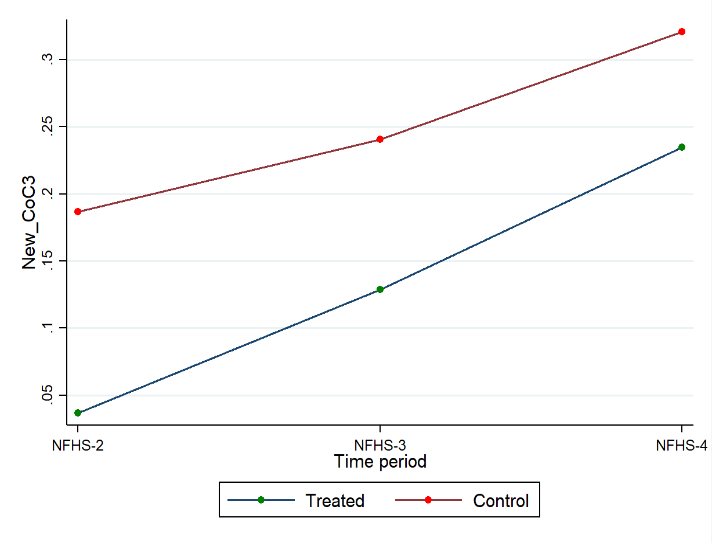
*

(c)

**Notes:** This figure illustrates trends in access to the continuum of care among treated and control women across NFHS-2, NFHS-3, and NFHS-4. The treated group comprises women eligible for the JSY program; women who were not eligible constitute the control group. CoC1, CoC2, and New_CoC3 are defined as follows: CoC1 – women who had uptake of at least three antenatal care (ANC) check-ups; CoC2 – CoC1 plus institutional delivery; and New_CoC3 – CoC2 plus the child received full immunization (one dose of BCG, three doses each of DPT and polio vaccines, and one dose of measles vaccine). Source: Authors’ calculations using NFHS-2, NFHS-3, and NFHS-4.

*Table A1. Falsification test for the parallel trends assumption*

|  | CoC1 | | CoC2 | | CoC3 | | CoC4 | |
| --- | --- | --- | --- | --- | --- | --- | --- | --- |
|  | (1) | (2) | (3) | (4) | (5) | (6) | (7) | (8) |
| Eligible | 0.018 | 0.025 | -0.068*** | -0.062*** | -0.054*** | -0.058*** | -0.095*** | -0.087*** |
|  | (0.030) | (0.029) | (0.022) | (0.022) | (0.018) | (0.017) | (0.015) | (0.014) |
| False × Post1 | 0.004 |  | 0.004 |  | 0.004 |  | -0.039*** |  |
|  | (0.013) |  | (0.011) |  | (0.011) |  | (0.010) |  |
| Eligible × False Post1 | 0.016 |  | 0.011 |  | -0.001 |  | 0.083*** |  |
|  | (0.013) |  | (0.010) |  | (0.010) |  | (0.009) |  |
| False × Post2 |  | 0.003 |  | -0.003 |  | -0.000 |  | -0.141*** |
|  |  | (0.011) |  | (0.010) |  | (0.009) |  | (0.009) |
| Eligible × False Post2 |  | 0.012 |  | 0.004 |  | 0.003 |  | 0.140*** |
|  |  | (0.013) |  | (0.010) |  | (0.008) |  | (0.009) |
|  |  |  |  |  |  |  |  |  |
| Controls | Yes | Yes | Yes | Yes | Yes | Yes | Yes | Yes |
| Birth year FE | Yes | Yes | Yes | Yes | Yes | Yes | Yes | Yes |
| State FE | Yes | Yes | Yes | Yes | Yes | Yes | Yes | Yes |
| Observations | 21,216 | 21,216 | 21,273 | 21,273 | 21,288 | 21,288 | 21,210 | 21,210 |
| R-squared | 0.395 | 0.395 | 0.437 | 0.437 | 0.403 | 0.403 | 0.250 | 0.265 |

**Notes:** This table presents intent-to-treat (ITT) estimates from a falsification test using NFHS-2 data. The variables False Post1 and False Post2 take the value 1 for children born after April 1997 and April 1998, respectively, simulating treatment periods that precede the actual program implementation. Eligible denotes women who would have been eligible for the program based on its eventual criteria. The outcome variables represent different stages of the continuum of care, defined as follows: CoC1 – uptake of at least three antenatal care (ANC) check-ups; CoC2 – CoC1 plus institutional delivery; CoC3 – CoC2 plus postnatal care (PNC) within two months of childbirth; and CoC4 – CoC3 plus the child received full immunization (one dose of BCG, three doses each of DPT and polio vaccines, and one dose of measles vaccine). The models control for individual characteristics, including the mother’s age, age at first birth, number of children ever born, years of education, caste, religion, residential location (rural/urban), household wealth, and sex of the household head. They also include the child’s birth year and state fixed effects. Standard errors are clustered at the PSU level and reported in parentheses. ***p < 0.01, **p < 0.05, *p < 0.1. Source: Authors’ calculations using NFHS-2.

*Table A2. Effect of JSY on maternal and child health services*

|  | ANC | Int. Delivery | PNC | FIMM |
| --- | --- | --- | --- | --- |
|  | 1 | 2 | 3 | 4 |
|  |  |  |  |  |
| Eligible | -0.236*** | -0.145*** | -0.154*** | -0.203*** |
|  | (0.010) | (0.010) | (0.010) | (0.008) |
| Post | 0.149*** | 0.354*** | 0.269*** | -0.482*** |
|  | (0.010) | (0.012) | (0.013) | (0.010) |
| Eligible × Post | 0.244*** | 0.175*** | 0.171*** | 0.219*** |
|  | (0.010) | (0.010) | (0.010) | (0.008) |
|  |  |  |  |  |
| Controls | Yes | Yes | Yes | Yes |
| State FE | Yes | Yes | Yes | Yes |
| Birth Year FE | Yes | Yes | Yes | Yes |
| Observations | 119,708 | 120,513 | 88,735 | 115,748 |
| R-squared | 0.207 | 0.300 | 0.180 | 0.198 |

**Notes:** The table presents intent-to-treat (ITT) estimates. ANC indicates whether a woman had at least one antenatal care visit. Int. Delivery is a binary variable equal to 1 if the woman delivered in a health facility. PNC equals 1 if the woman received a postnatal check-up within two months of delivery. FIMM equals 1 if the child received one dose of BCG, three doses each of DPT and polio vaccines, and one dose of the measles vaccine. Eligible denotes women eligible for the program. Post is a binary variable equal to 1 for births occurring in the post-program launch period. The models control for individual characteristics, including the mother’s age, the number of children ever born, years of education, caste, religion, residential location (rural/urban), and household characteristics, such as wealth and the sex of the household head. They also include the child’s birth year and state fixed effects. Standard errors are clustered at the PSU level and reported in parentheses. *** p<0.01, ** p<0.05, * p<0.1. Source: Authors’ calculations using NFHS-2 and NFHS-4.

*Table A3. Impact of JSY on the continuum of maternal and child health care in low-performing states*

|  | CoC1 | CoC2 | CoC3 | CoC4 |
| --- | --- | --- | --- | --- |
|  | (1) | (2) | (3) | (4) |
|  |  |  |  |  |
| Eligible | -0.469*** | -0.331*** | -0.230*** | -0.075*** |
|  | (0.013) | (0.016) | (0.015) | (0.010) |
| Post | 0.216*** | 0.339*** | 0.215*** | -0.113*** |
|  | (0.012) | (0.011) | (0.011) | (0.008) |
| EligLPS × Post | 0.123*** | 0.027*** | 0.053*** | 0.047*** |
|  | (0.009) | (0.009) | (0.008) | (0.006) |
|  |  |  |  |  |
| Controls | Yes | Yes | Yes | Yes |
| State FE | Yes | Yes | Yes | Yes |
| Birth Year FE | Yes | Yes | Yes | Yes |
| Observations | 102,115 | 102,278 | 86,441 | 94,142 |
| R-squared | 0.242 | 0.279 | 0.212 | 0.122 |

**Notes:** The table presents intent-to-treat (ITT) estimates. Columns (1)–(4) correspond to successive stages of the maternal and child health (MCH) continuum of care (CoC1–CoC4) as dependent variables. CoC1–CoC4 are defined as follows: CoC1 – women with uptake of at least three antenatal care (ANC) check-ups; CoC2 – CoC1 plus institutional delivery; CoC3 – CoC2 plus postnatal care (PNC) within two months of childbirth; and CoC4 – CoC3 plus the child received full immunization (one dose of BCG, three doses each of DPT and polio vaccines, and one dose of measles vaccine). *EligLPS* takes the value 1 for women eligible under the program in low-performing states and 0 for non-eligible women. *Post* is a binary indicator equal to 1 for births occurring in the post-program launch period. The models control for individual characteristics, including the mother’s age, age at first birth, number of children ever born, years of education, caste, religion, and residential location (rural/urban), as well as household characteristics such as wealth and the sex of the household head. They also include the child’s birth year and state fixed effects. Standard errors are clustered at the PSU level and reported in parentheses. *** p<0.01, ** p<0.05, * p<0.1. Source: Authors’ calculations using NFHS-2 and NFHS-4.

*Table A4. Effect of JSY on the continuum of maternal and child health care in high-performing states*

|  | CoC1 | CoC2 | CoC3 | CoC4 |
| --- | --- | --- | --- | --- |
|  | (1) | (2) | (3) | (4) |
|  |  |  |  |  |
| Eligible | -0.018 | -0.038* | -0.053*** | -0.034** |
|  | (0.029) | (0.022) | (0.019) | (0.015) |
| Post | 0.120*** | 0.277*** | 0.165*** | -0.183*** |
|  | (0.015) | (0.015) | (0.014) | (0.012) |
| EligHPS × Post | 0.018 | 0.058** | 0.073*** | 0.039** |
|  | (0.029) | (0.023) | (0.021) | (0.015) |
|  |  |  |  |  |
| Controls | Yes | Yes | Yes | Yes |
| State FE | Yes | Yes | Yes | Yes |
| Birth Year FE | Yes | Yes | Yes | Yes |
| Observations | 46,994 | 47,083 | 36,114 | 41,324 |
| R-squared | 0.237 | 0.322 | 0.259 | 0.160 |

**Notes:** The table presents intent-to-treat (ITT) estimates. Columns (1)–(4) correspond to successive stages of the maternal and child health (MCH) continuum of care (CoC1–CoC4) as dependent variables. CoC1–CoC4 are defined as follows: CoC1 – uptake of at least three antenatal care (ANC) check-ups; CoC2 – CoC1 plus institutional delivery; CoC3 – CoC2 plus postnatal care (PNC) within two months of childbirth; and CoC4 – CoC3 plus the child received full immunization (one dose of BCG, three doses each of DPT and polio vaccines, and one dose of measles vaccine). EligHPS takes the value 1 for women eligible under the program in high-performing states and 0 for non-eligible women. Post is a binary indicator equal to 1 for births occurring in the post-program launch period. The models control for individual characteristics, including the mother’s age, age at first birth, number of children ever born, years of education, caste, religion, and residential location (rural/urban), as well as household characteristics such as household wealth and sex of the household head. They also include the child’s year of birth and state fixed effects. Standard errors are clustered at the PSU level and reported in parentheses. *** p<0.01, ** p<0.05, * p<0.1. Source: Authors’ calculations using NFHS-2 and NFHS-4.

*Table A5. Effect of JSY on the continuum of care: Propensity score matching (PSM) analysis*

|  | CoC1 | CoC2 | CoC3 | CoC4 |
| --- | --- | --- | --- | --- |
|  | (1) | (2) | (3) | (4) |
|  |  |  |  |  |
| Eligible | -0.114*** | -0.002 | -0.025*** | -0.039*** |
|  | (0.009) | (0.009) | (0.008) | (0.006) |
| Post | 0.209*** | 0.336*** | 0.216*** | -0.114*** |
|  | (0.011) | (0.011) | (0.011) | (0.008) |
| Eligible × Post | 0.121*** | 0.027*** | 0.050*** | 0.046*** |
|  | (0.009) | (0.009) | (0.008) | (0.006) |
|  |  |  |  |  |
| Controls | Yes | Yes | Yes | Yes |
| State FE | Yes | Yes | Yes | Yes |
| Birth Year FE | Yes | Yes | Yes | Yes |
| Observations | 119,534 | 119,741 | 98,357 | 108,694 |
| R-squared | 0.253 | 0.293 | 0.217 | 0.124 |

**Notes**: This table presents intent-to-treat (ITT) estimates on the matched sample using propensity score matching (PSM). Columns (1)–(4) correspond to successive stages of the maternal and child health (MCH) continuum of care (CoC1–CoC4) as dependent variables. CoC1–CoC4 are defined as follows: CoC1 – uptake of at least three antenatal care (ANC) check-ups; CoC2 – CoC1 plus institutional delivery; CoC3 – CoC2 plus postnatal care (PNC) within two months of childbirth; and CoC4 – CoC3 plus the child received full immunization (one dose of BCG, three doses each of DPT and polio vaccines, and one dose of measles vaccine). *Eligible* denotes women eligible for the program. *Post* is a binary variable equal to 1 for births occurring in the post-program launch period. The models control for individual characteristics, including the mother’s age, age at first birth, number of children ever born, years of education, caste, religion, and residential location (rural/urban), as well as household characteristics such as wealth and the sex of the household head. They also include the child’s birth year and state fixed effects. Standard errors are clustered at the PSU level and reported in parentheses. *** p<0.01, ** p<0.05, * p<0.1. Source: Authors’ calculations using NFHS-2 and NFHS-4.

*Table A6. Effect of JSY on the continuum of maternal and child health care with NFHS-3 & NFHS-4*

|  | CoC1 | | CoC2 | | New_CoC3 | |
| --- | --- | --- | --- | --- | --- | --- |
|  | (1) | (2) | (3) | (4) | (5) | (6) |
|  |  |  |  |  |  |  |
| Eligible | -0.005 | -0.023*** | 0.011 | -0.010 | 0.014* | 0.001 |
|  | (0.009) | (0.007) | (0.010) | (0.007) | (0.007) | (0.006) |
| Post | 0.086*** | 0.121*** | 0.224*** | 0.266*** | -0.243*** | -0.200*** |
|  | (0.012) | (0.012) | (0.014) | (0.011) | (0.011) | (0.010) |
| Eligible × Post | 0.055*** | 0.040*** | 0.053*** | 0.034*** | 0.027*** | 0.014** |
|  | (0.010) | (0.008) | (0.011) | (0.007) | (0.008) | (0.006) |
|  |  |  |  |  |  |  |
| Controls | No | Yes | No | Yes | No | Yes |
| State FE | Yes | Yes | Yes | Yes | Yes | Yes |
| Birth Year FE | Yes | Yes | Yes | Yes | Yes | Yes |
| Observations | 174,186 | 164,622 | 174,569 | 164,988 | 172,782 | 163,301 |
| R-squared | 0.165 | 0.255 | 0.163 | 0.294 | 0.141 | 0.209 |

**Notes:** This table presents intent-to-treat (ITT) estimates. Columns (1)–(6) report results for successive stages of the maternal and child health (MCH) continuum of care (CoC1, CoC2, and New_CoC3) as dependent variables. CoC1 is defined as uptake of at least three antenatal care (ANC) check-ups; CoC2 as CoC1 plus institutional delivery; and New_CoC3 as CoC2 plus the child receiving full immunization (one dose of BCG, three doses each of DPT and polio vaccines, and one dose of measles vaccine). Eligible denotes women eligible under the program. Post is a binary variable equal to 1 for births occurring in the post-program launch period. The models control for individual characteristics, including the mother’s age, age at first birth, number of children born, years of education, caste, religion, and residential location (rural/urban), as well as household characteristics such as household wealth and sex of the household head. They also include the child’s birth year and state fixed effects. Standard errors are clustered at the PSU level and reported in parentheses. *** p<0.01, ** p<0.05, * p<0.1. Source: Authors’ calculations using NFHS-3 and NFHS-4.
